# The impact of teacher feedback on students’ decisions to stay on or change course after math failure in a Confucian cultural context

**DOI:** 10.3389/fpsyg.2022.1046806

**Published:** 2022-12-07

**Authors:** Bih-Jen Fwu, Tong-Rong Yang, Yi-Kai Chen, Rong Chen

**Affiliations:** ^1^Center for Teacher Education, National Taiwan University, Taipei, Taiwan; ^2^Department of Psychology, National Taiwan University, Taipei, Taiwan; ^3^Department of Psychology, Dominican University of California, San Rafael, CA, United States

**Keywords:** entity belief, math failure, obligation belief, path-changing, teacher feedback

## Abstract

Previous research indicated that instructors holding entity belief tended to judge students to have low ability and provided ability-comforting feedback following math failure. Students receiving such feedback tended to quit and change course, creating a potential decrease in the pool of students pursuing math related fields. In Confucian heritage cultures (CHCs), the ideal society is primarily based on fulfillment of duties. Thus, the ability-based findings, derived from WEIRD samples, may not apply to duty-based CHCs. We hypothesized that CHC’s teachers holding obligation belief tend to attribute students’ failure to lack of duty fulfillment and provide duty-based feedback, including duty-comforting and duty-advising feedback, which motivates students to stay on rather than change course. To validate our hypothesis, we conducted three scenario experiments with 160 college students with teaching experiences, 273 high school students, and 369 pre-service teachers in Taiwan. Results showed that while ability-based paradigm may be culture-free, duty-based paradigm seems to be culture-bound. Consistent with previous research, teachers with entity belief tended to give ability-comforting feedback, pushing students to pursue non-math related fields. In contrast, teachers with obligation belief were likely to offer duty-comforting and duty-advising feedback, contributing to students’ persistent pursuit in math. Furthermore, three fifths of teachers were inclined to provide ability-comforting, duty-comforting and duty-advising feedback concurrently, thus putting students in an unpleasant predicament that might be detrimental to their psychological well-being. Theoretical and practical implications are discussed.

## Introduction

Failure is an inevitable yet essential part of learning. Students’ decision to persist or quit after setbacks is likely influenced by teacher feedback, which has been identified as one of the top ten factors that affect student achievement (Hattie, 2009). Teacher feedback may inadvertently convey teacher’s belief and attribution, which impact student motivation. Rattan et al. (2012) found that instructors endorsing an entity theory tended to judge students to have low ability thus comfort students who failed math by saying “It’s OK. It’s just not the case that everyone is a ‘math person.’ I want you to remember how great you do in other subjects.” Such ability-comforting feedback demotivated students to learn math, and rather motivated them to pursue non-math related fields, creating a potential crisis of shrinking STEM (Science, Technology, Engineering, and Math) talent pool.

Prior studies have examined the sociocultural influences on student approaches to learning (Biggs, 1996), students’ attitudes towards assessment (Kennedy et al., 2008; Leong et al., 2018), and students’ attitudes toward mathematics in flipped classrooms (Ryan et al., 2014; Karjanto and Simon, 2019). By the same token, studies have emphasized the importance of understanding the role of teacher feedback in student learning in school settings within their own sociocultural context (de Luque and Sommer, 2000; Torrance, 2012; Yang and Yang, 2018). Many researchers (Arnett, 2008; Henrich et al., 2010; Raffaelli et al., 2013; Pollet and Saxton, 2019) have raised concerns that a large majority of psychological research came from WEIRD (Western, Educated, Industrialized, Rich, and Democratic) samples, predominantly American undergraduates, who only represent 12% of the world population. However, most non-WEIRD scholars assumed that these research findings represent “truth” and implanted them to their native countries unquestionably (Hwang, 2010), which may cause neglect of important cultural factors that have profound impacts on human behavior in their own cultures (Hwang, 2005). In Confucian heritage cultures (CHCs), the ideal society is primarily based on fulfillment of duties (Cheng, 1990). Therefore, Rattan et al.’s ability-based findings, derived from WEIRD samples, may not fully apply to duty-based CHCs. We argue that CHC’s teachers tend to provide duty-based feedback, which motivates students to stay on rather than change course. Such persistence to overcome learning difficulties and challenges may lead students to perform well in international assessments such as TIMSS and PISA (Schleicher, 2019; Mullis et al., 2020) and provide abundant talent pool in STEM (Guo, 2013).

Li (2002, 2005, 2012) indicated that Western and CHC’s people hold fundamentally different beliefs about learning that influence how they approach education. The Western mind-oriented model of learning focuses on cognitive domain, aiming to cultivate the mind to understand the world, whereas the CHC’s virtue-oriented model of learning stresses the development of the whole person, intending to perfect oneself morally and maintain harmonious relationships with significant others socially. In line with mind-oriented model, the aforementioned ability-based feedback seems to be related to Dweck’s entity theory (Dweck and Leggett, 1988; Dweck, 1999), focusing on an individual’s belief that human traits and qualities such as abilities are fixed. Previous research indicated that teacher emotions and behaviors, including ability-comforting feedback, may unintentionally and unwittingly communicate low-ability cues (Graham, 1984; Barker and Graham, 1987; Graham and Barker, 1990; Butler, 1994; Georgiou et al., 2002; Weiner, 2010; Rattan et al., 2012; Lou and Noels, 2020; Taxer and Frenzel, 2020). For entity theorists, if perceived ability to perform a task is low, the perceived possibility for mastery and success is also low. They thus tend to pursue a task they are good at because they have a better chance of success.

In accordance with virtue-oriented model, duty-based feedback appears to be associated with obligation belief, which can be dated back to the role obligation theory of self-cultivation (ROT) in CHCs (Fwu et al., 2021). The ROT perspective argues that individuals’ traits *should* be continuously improved to achieve the ultimate good (zhì shàn, 至善), as depicted in a Chinese axiom, “If you can improve yourself in a day, do so each day, forever building on improvement” (original source: 《dàxué》gǒu rì xīn rì rì xīn yòu rì xīn, 《大學》「茍日新，日日新，又日新」) (Fwu et al., 2021). Hence, obligation belief emphasizes that continual and endless self-improvement is not only possible but also mandatory. It is one’s *obligation* to realize one’s full potential through a continuous process of self-perfection to become an ideal virtuous person (Hwang, 2012; Fwu et al., 2017). As expectations for achieving the ultimate good can be elevated without limits, one should perfect oneself to fulfill one’s duty in one’s lifetime. The obligation belief appears to corroborate to incremental belief, i.e., the opposite of entity belief, because it stresses that human attributes are not fixed.

In school settings, CHC’s students are expected to work hard and perform well so as to fulfill their role obligations. Research conducted in CHCs indicated that schoolteachers gave struggling students duty-based feedback (Wang, 1998). For those who were slow but fulfilled their duty as hardworking students, teachers tended to offer duty-comforting feedback, like “It’s OK. As long as you have exerted yourself in the learning process, you do not owe anyone an apology.” For those who were smart but did not work hard to fulfill their duty, teachers tended to give duty-advising feedback by saying “You reap what you sow. If you try your hardest, you will certainly perform better” (Wang, 1998). While duty-comforting feedback focuses on consoling students for hard work and suggesting working smart strategy to perform well, duty-advising feedback stresses advising students to cultivate their moral virtues by working hard. Thanh Pham and Renshaw (2015) contend that the cultural values internalized through students’ socialization heavily influence the ways in which they perceive and respond to teacher feedback.

Taken together, we hypothesized that consistent with Rattan et al.’s findings, teachers holding entity belief are likely to judge students to have low ability and give ability-comforting feedback after math failure, whereas those holding obligation belief tend to diagnose students’ lack of duty fulfillment and provide duty-based feedback (H1). Furthermore, students receiving ability-comforting feedback are inclined to pursue non-math related fields, whereas those receiving duty-based feedback tend to persist in math (H2). Lastly, cross-cultural research has indicated that North Americans who succeeded on a task persisted on a follow-up task, whereas East Asian students tended to persist after failure (Heine et al., 2001; Zhang and Cross, 2011). Consequently, we hypothesized that CHC’s teachers tend to provide duty-comforting and duty-advising feedback that motivate students to stay on rather than change course (H3). We tested these hypotheses in three studies. This research has obtained IRB approval from the Institutional Review Board at National Taiwan University (NTU-REC: 201705HS032 & 201805HS009).

## Study 1

The aim of Study 1 was to examine whether Rattan et al.’s (2012) claim that instructors holding entity belief were more likely to make low ability attribution and give ability-comforting feedback is culture-free. Moreover, we explored if teachers holding culturally relevant obligation belief tended to ascribe failure to insufficient duty fulfillment and provide duty-comforting and duty-advising feedback, signifying a culture-bound phenomenon. We investigated the impacts of teacher beliefs on the attributions of student failure and the types of feedback given to failing students.

### Method

#### Participants and procedure

Participants were 160 college students with teaching experience in their own study fields from a top university in Northern Taiwan. Data collection occurred during TA workshops or regular classes. Trained research assistants administered the questionnaire. After giving their consent, participants responded to the questionnaire on a voluntary and anonymous basis. All participants received NTD $50 (approximately USD $2) in compensation for their time. To ensure data quality, 2 participants were excluded for failing attention checks, resulting in a valid sample size of 158 (98 males, *M*_age_ = 23.25, *SD* = 2.94; 60 females, *M*_age_ = 21.18, *SD* = 2.83).

We applied the scenario method, which may be like a behavioral observation in controlled conditions where unwanted situational factors are minimized (Peng et al., 1997). Participants began with a “teacher belief” survey, in which entity and obligation beliefs were embedded. Participants were then instructed to read a scenario that “after the mid-term examination, you met with a student named Minghua (明華, gender neutral name in CHCs), who had received 65 out of 100 points on the first test of the year, and discussed their performance individually.” Next, participants were asked about their attribution to Minghua’s math performance and a series of feedback items measuring the degree to which they would give to them.

#### Measures

All measures outlined below were rated on a Likert-type scale ranging from 1 (*strongly disagree*) to 6 (*strongly agree*). All detailed items of each construct in teacher belief, attribution and feedback were listed in Supplementary material 1.

##### Teacher belief

Teacher belief included entity belief and obligation belief.

*Entity belief (EB)* meant viewing personality trait/quality as an unchangeable, fixed internal characteristic, which was measured by four items adopted from “Kind of Person” Implicit Theory Scale (Dweck, 1999; *α* = 0.88). A sample item from the scale read, “Everyone is a certain kind of person, and there is not much that can be done to really change that.” Higher scores indicate higher tendency of holding entity belief.

*Obligation belief (OB)* was defined as viewing improving one’s personality trait/quality as one’s own duty and responsibility, which was measured by five items derived from the Scale of Role Obligation Theory of Self-cultivation (Yang et al., 2021a; *α* = 0.90). A sample item from the scale read, “One should be ever-seeking to improve and further refine oneself.” Higher scores indicate higher tendency of holding obligation belief.

##### Teacher attribution

Teacher attribution referred to how participants explained the causes of the protagonist’s academic failure in the scenario, which was measured by two factors: Minghua’s performance was not satisfactory because of a lack of ability and a lack of duty fulfillment.

*Lack of ability (LA)* was defined as low inborn talent, which was assessed by three items taken from the Scale of Failure Attribution (Yang et al., 2021b; *α* = 0.88). A sample item from the scale read, “Minghua is not good at the subject.” Higher scores indicate higher level of low ability.

*Lack of duty fulfillment (LD)* referred to failing to exert himself/herself to fulfill his/her duty, which was measured by four items taken from the Scale of Failure Attribution (Yang et al., 2021b; *α* = 0.90). A sample item from the scale read, “Minghua did not do whatever he/she can to fulfill his/her obligation.” Higher scores indicate higher level of insufficient duty fulfillment.

##### Teacher feedback

Teacher feedback referred to information provided by teachers following a student’s math failure, including ability-comforting, duty-comforting and duty-advising feedback.

*Ability-comforting feedback (AC)* was defined as consoling struggling students for their low ability and enacting potentially unhelpful pedagogical practices, which were assessed by eight items adapted from Rattan et al.’s (2012) research (Chen et al., 2021; *α* = 0.82). Two sample items from the scale included, “It’s just not the case that everyone has a talent for this subject” and “I’m going to give you some easier tasks to work on so you can get more comfortable with those skills.” Higher scores indicate higher likelihood of giving ability-comforting feedback.

*Duty-comforting feedback (DC)* was defined as consoling failing students’ disappointment by praising their good learning attitudes and offering working-smart strategies for success, which were assessed by seven items adapted from Chen et al.’s (2021) research (α = 0.82). Two sample items from the scale included, “Good attitude in the learning process is more important than end results” and “If you have trouble understanding what was taught, you can ask your classmates for help.” Higher scores indicate higher likelihood of giving duty-comforting feedback.

*Duty-advising feedback (DA)* was defined as a reminder of “no pains, no gains” and giving working-hard strategies for success, which were assessed by eight items adapted from Wang’s (1998) research (Chen et al., 2021; *α* = 0.89). Two sample items from the scale included, “You reap what you sow” and “I suggest you review what was taught in class.” Higher scores indicate higher likelihood of giving duty-advising feedback.

### Results and discussion

To validate H1, we identified participants with low-and high-scores on entity belief and obligation belief, respectively. Participants with high and low scores were defined as participants whose scale average value was one standard deviation above and below the scale mean, respectively. Table 1 listed Means, SD, cut-off values, and numbers of students in low-score and high-score groups in entity and obligation beliefs.

**Table 1:** Means, SD, cut-off values, and numbers of low-and high-score groups of EB and OB.

	Mean	SD	Cut-off values of low-score group	Cut-off values of high-score group	Numbers of participants in low-score group	Numbers of participants in high-score group
EB	3.30	1.09	2.21	4.39	21	21
OB	4.48	0.96	3.52	5.44	20	26

EB, Entity belief; OB, Obligation belief.

**Figure 1:**
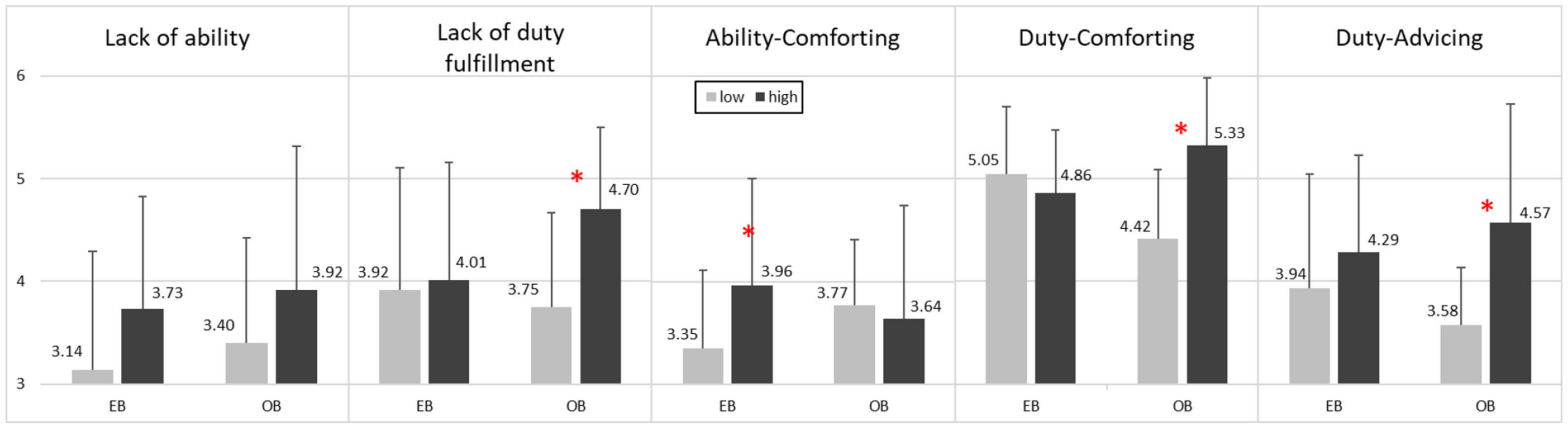
Mean scores of lack of ability, lack of duty fulfillment, ability-comforting, duty-comforting, and duty-advising feedback between low-and high-score groups in EB and OB. EB, Entity belief; OB, Obligation belief. Scores between low-and high-score of EB and OB groups were compared using two tailed *t* test. *p < 0.05.

Unexpectedly, results indicated that there was no significant difference in lack of ability between high-score and low-score groups of entity belief. However, as hypothesized, results indicated a significant difference in lack of duty fulfillment between high-score (*M* = 4.70, *SD* = 0.80) and low-score groups (*M* = 3.75, *SD* = 0.92) of obligation belief, *t*(44) = 3.20, *p* < 0.05, but no significant difference between the two groups of entity belief. Moreover, as predicted, we found a significant difference in ability-comforting feedback between high-score (*M* = 3.96, *SD* = 1.04) and low-score groups (*M* = 3.35, *SD* = 0.76) of entity belief, *t*(83) = 2.20, *p* < 0.05, but no significant difference between the two groups of obligation belief. We also found a significant difference in duty-comforting feedback between high-score (*M* = 5.33, *SD* = 0.66) and low-score groups (*M* = 4.42, *SD* = 0.67) of obligation belief, *t*(44) = 4.86, *p* < 0.05, but no difference between the two groups of entity belief. Similarly, there was a significant difference in duty-advising feedback between high-score (*M* = 4.57, *SD* = 1.16) and low-score groups (*M* = 3.58, *SD* = 0.56) of obligation belief, *t*(44) = 4.51, *p* < 0.05, but no significant difference between the two groups of entity belief (see Figure 1).

In sum, results replicated Rattan et al.’s (2012) findings that instructors who held entity belief were inclined to provide ability-comforting feedback. In contrast, instructors who held obligation belief tended to ascribe failure to lack of duty fulfillment and offered duty-comforting and duty-advising feedback.

How would students perceive their teacher’s belief and attribution and respond after receiving three different types of teacher feedback following math failure? Study 2 explored these questions through three scenarios associated with ability-comforting, duty-comforting and duty-advising feedback conditions and examined students’ perceptions and decisions to stay on or change course after hearing such feedback from a teacher.

## Study 2

The aim of Study 2 was to investigate the differences in students’ perceptions of teacher belief and attribution as well as their own decisions to change course or stay on, among students exposed to ability-comforting, duty-comforting and duty-advising feedback conditions following math failure.

### Method

#### Participants and procedure

Rattan et al. (2012) conducted research at a competitive university in North America where students do not have to declare major until enrolling in universities. However, senior high school students in Taiwan need to select a major of study: STEM or humanities/social sciences by 10^th^ grade, which determines their majors in university. Moreover, while most public senior high schools are coeducational, major public “star” senior high schools are single-gender. In an effort to replicate Rattan et al.’s (2012) research, we recruited 273 tenth graders from two highly selective single-sex senior high schools, one for boys and the other for girls in the Taipei metropolitan area, which admitted students who scored top on national entrance examinations. Data collection occurred a week after mid-term math examinations during regular classes. Trained research assistants administered the questionnaire. After giving their consent, participants responded to the questionnaire on a voluntary and anonymous basis. All participants received NTD $50 (approximately USD $2) in compensation for their time. To ensure data quality, 3 participants were excluded because of failed attention checks, resulting in a valid sample size of 270 (126 boys, *M*_age_ = 15.89, *SD* = 0.34; 144 girls, *M*_age_ = 15.87, *SD* = 0.41).

We applied the scenario method that described “after the mid-term math examination, you met with your teacher to learn your low score on the math test (65 out of 100 points). Your math teacher noticed that you were not happy and probably disappointed by your grade.” Participants were then randomly assigned to one of the three hypothetical feedback conditions, including ability-comforting, duty-comforting, and duty-advising feedback. Ability-comforting feedback focused on students’ strengths and comforting their weaknesses. Duty-comforting feedback emphasized praising students’ good learning attitudes and offering strategies for studying smarter, whereas duty-advising feedback stressed urging students to study hard to fulfill their duties. All detailed conditions of teacher feedback were listed in Supplementary material 2. Participants pretended that the hypothetical situation happened to them. After reading the scenario, participants were asked to respond to 22 items rated on a Likert-type scale ranging from 1 (*strongly disagree*) to 6 (*strongly agree*). Their responses indicated how they personally felt about the feedback at that time.

#### Measures

The response items of each construct, including perceived teacher belief, perceived teacher attribution, and students’ behavioral intentions were listed in Supplementary material 1.

##### Perceived teacher belief

Perceived teacher belief referred to participants’ perceptions about their teacher belief, including perceived entity belief and perceived obligation belief.

*Perceived entity belief (EBp)* meant that participants viewed their teachers as having entity belief, which was measured by four items taken from the “Kind of Person” Implicit Theory Scale (Dweck, 1999; e.g., “I felt my teacher believed everyone is a certain kind of person, and there is not much that can be done to really change that,” *α* = 0.90). Higher scores indicate higher levels of perceived teacher’s entity belief.

*Perceived obligation belief (OBp)* meant that participants viewed their teachers as having obligation belief, which was assessed by four items from the Obligation Belief Scale (Yang et al., 2021a; e.g., “I felt my teacher believe one should be ever-seeking to improve and further refine oneself,” *α* = 0.94). Higher scores indicate higher levels of perceived teacher’s obligation belief.

##### Perceived teacher attribution

Perceived teacher attribution referred to participants’ perceptions about teacher’s failure attributions, including perceived lack of ability and perceived lack of duty fulfillment.

*Perceived lack of ability (LAp)* meant that participants regarded their teachers as attributing failure to low innate talent, which was measured with three items from the Scale of Failure Attribution to Lack of Ability (Yang et al., 2021b; e.g., “I felt my teacher think I’m not good at math,” *α* = 0.94). Higher scores represent higher levels of perceived teacher’s low ability attribution.

*Perceived lack of duty fulfillment (LDp)* meant that participants viewed their teachers as attributing failure to insufficient duty fulfillment, which was assessed with four items from the Scale of Failure Attribution to Lack of Duty Fulfillment (Yang et al., 2021b; e.g., “I felt my teacher think I did not do my best to fulfill my role as a student,” *α* = 0.96). Higher scores represent higher levels of perceived teacher’s low duty fulfillment attribution.

##### Students’ behavioral intentions

Behavioral intentions referred to participants’ decisions to stay on or change path. Rattan et al. (2012) indicated that participants in ability-comforting feedback are unmotivated to work on math, and might pursue non-math related areas. This study, aside from persistent behavior in math following failure, added path-changing to directly assess participants’ decision to switch to non-math related fields.

*Staying-on* referred to the intention of persistence to study math, which was measured with three items adapted from Rattan et al. (2012) and Fwu et al. (2018) (e.g., “I do not give up easily in the face of difficulty on math tests,” *α* = 0.80). Higher scores indicate higher intention of persisting in math.

*Path-changing* meant the intention to pursue a non-math field, which was assessed with four items from the Scale of Path-Changing (Chen et al., 2021; e.g., “I will switch to a field that I am good at,” *α* = 0.91). Higher scores indicate higher intention of changing to non-math fields.

### Results and discussion

#### Profile analysis

**Figure 2:**
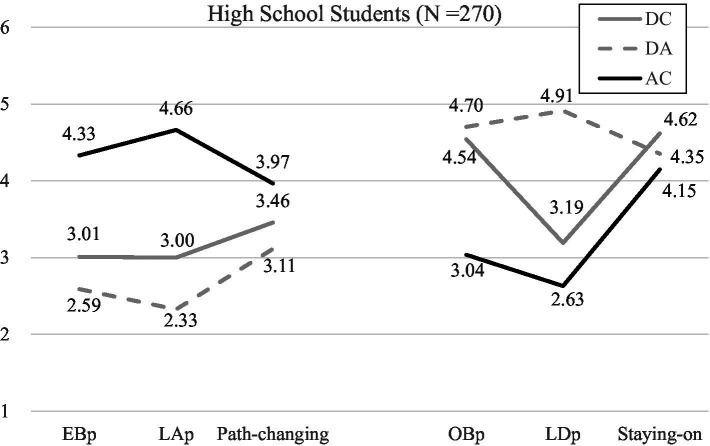
Results of profile analysis of the impact of teacher feedback on students’ perceived teacher belief, teacher attribution, and students’ behavioral intentions. EBp, Perceived entity belief; LAp, Perceived lack of ability; OBp, Perceived obligation belief; LDp, Perceived lack of duty fulfillment.

Descriptive statistics for all items for high-school students were shown in Supplementary material 3. To validate H2, profile analysis was used to identify whether three groups of participants in this study show a significantly distinct profile. As expected and shown in Figure 2, students in an ability-comforting feedback condition were most likely not only to perceive their teacher as endorsing entity belief and attributing failure to low ability, but also to pursue non-math fields among the three feedback conditions. In contrast, students exposed in duty-based conditions, including both duty-comforting and duty-advising feedback, were more inclined not only to perceive their teacher as having obligation belief and attributing failure to insufficient duty fulfillment, but also to stay on math field than those in the ability-comforting feedback condition. There were no significant differences between girls and boys in their perception of teacher belief, teacher attribution, and their own behavioral intentions.

In short, consistent with Rattan et al.’s (2012) research, ability-comforting feedback not only conveyed more of an entity theory and a cue of lack of ability, but also led students to pursue non-math related fields compared to the other two duty-based feedback conditions. Conversely, duty-comforting and duty-advising feedback not only reflected more of an obligation belief and a cue of lack of insufficient duty fulfillment, but also motivated students to persist in math learning. Moreover, it is worth noting that, both ability-comforting and duty-comforting feedback attempted to ease students’ discomfort and pressure following math failure; however, the former pushed students to change to non-math fields, whereas the latter encouraged them to stay on.

Study 2 indicated that while ability-comforting feedback led students to switch to non-math related fields, duty-comforting and duty-advising feedback led students to persist in math. How would CHC’s teacher give feedback to students following academic failure? How can teachers be categorized by using the aforementioned feedback conditions (i.e., ability-comforting, duty-comforting, and duty-advising)? This question was addressed in the next study.

## Study 3

The aim of Study 3 was to investigate whether a majority of teachers tend to provide duty-based feedback rather than ability-comforting feedback, thus motivating failing students to stay on.

### Method

#### Participants and procedure

A total of 369 pre-service teachers of secondary education programs were recruited from three universities in Northern Taiwan. Data collection occurred during regular classes. Trained research assistants administered the questionnaire. After giving their consent, participants responded to the questionnaire on a voluntary and anonymous basis. Each participant was compensated NTD $50 for their participation. Three participants were excluded for failing the attention check questions, resulting in a valid sample size of 366 (178 males, *M*_age_ = 21.63, *SD* = 3.09; 188 females, *M*_age_ = 21.90, *SD* = 3.50). The sample included 38.3% STEM majors, 41.3% Humanities/Social Science majors, and 20.5% other majors. We used the scenario method that depicted “you imagine yourself as a high school teacher. After the mid-term examination, you met with your student named Minghua, who had received 65 out of 100 points on the first test in the subject you taught. You had a chance to discuss with them individually.” Participants were asked to provide Minghua with feedback.

#### Measures

All measures for ability-comforting, duty-comforting, and duty-advising feedback were identical to Study 1.

### Results and discussion

#### Latent class analysis

To validate H3, we conducted a latent class analysis (LCA) to investigate possible underlying groups of teachers who gave feedback with the following three steps. First, to better explain the latent classes, we transferred the parceled indicators into 2-point scale before applying in LCA. Those above or equal to 4 points (4 = somewhat agree, 5 = agree, 6 = strongly agree) were coded as 1, whereas those below or equal to 3 (3 = somewhat disagree, 2 = disagree, 1 = strongly disagree) were coded as 0. Second, to find the most proper number of groups to explain the data, we took one to four latent class models into consideration and adopted the most interpretable and parsimonious model suggested by model fit indices, BIC and CAIC. Finally, according to the selected model, we explained the characteristics of each latent class. All data analysis procedures were performed in SAS (9.4; Lanza et al., 2007).

##### Model selection

Table 2 presents the model fit indices, of which two latent groups model with lowest BIC and CAIC (C2) were identified as the most fit.

**Table 2:** Model fit indices and degree of freedoms under models with different numbers of groups.

Models	C1	C2	C3	C4
BIC	287.78	163.02	184.74	180.4
CAIC	293.78	183.02	197.74	207.4
Entropy	1	0.69	0.77	0.77
df	57	50	43	36

##### Parameter estimates

Table 3 shows the results of LCA, indicating two major groups of teachers who gave different combinations of teacher feedback. A larger group was the combination of AC, DC and DA (60.0%), followed by a smaller group of combining DC and DA (40.0%).

**Table 3:** Conditional probabilities and group proportions of two observed latent groups.

	AC + DC + DA	DC + DA
proportion	60.0%	40.0%
AC1	**0.799**	0.053
AC2	**0.918**	0.201
DC1	**1.000**	**0.982**
DC2	**0.986**	**0.979**
DA1	**0.863**	**0.713**
DA2	**0.861**	**0.735**

Conditional probabilities greater than 0.700 were shown in bold. AC, Ability-comforting feedback; DC, Duty-comforting feedback; DA, Duty-comforting feedback. Detailed explanations of AC1, AC2, DC1, DC2, DA1, and DA2 were shown in Supplementary material 4.

In summary, unlike Rattan et al.’s (2012) findings, no teacher groups gave AC only. On the contrary, as expected, the two teacher groups provided duty-comforting and duty-advising feedback that motivate students to persist after academic failure. However, three-fifths of teachers gave ability-comforting, duty-comforting and duty-advising feedback concurrently. While ability-comforting feedback pushed struggling students to disengage from math learning, duty-comforting and duty-advising feedback encouraged them to stay on. Such mixed and contradictory feedback might create intra-personal conflicts and confusion for students of what to do next.

## General discussion

The present research intended to investigate the impacts of teacher feedback on students’ decision to stay on or change course after math failure in a Confucian cultural context. We found that, first, while teachers holding entity belief were inclined to provide ability-comforting feedback, those holding obligation belief tended to offered duty-comforting and duty-advising feedback. Second, ability-comforting feedback conveyed teacher’s entity belief and low ability attribution as well as led students to pursue non-math related fields, whereas duty-comforting and duty-advising feedback communicated teacher’s obligation belief and attribution to insufficient duty fulfillment as well as motivated students to persist in math learning. Third, both ability-comforting and duty-comforting feedback attempted to ease students’ discomfort and pressure following math failure; nevertheless, the former pushed students to pursue non-math fields, whereas the latter encouraged them to persist in math fields. Fourth, although all Taiwanese teachers tended to offer duty-comforting and duty-advising feedback, three-fifths of teachers gave ability-comforting, duty-comforting and duty-advising feedback concurrently. The theoretical significance and practical implications are discussed as follows.

### Culture-free vs. culture-bound in non-WEIRD societies

Arnett (2008) indicated that psychological research published in APA journals has been largely dominated by American researchers and subjects. Among first authors, 73% were based at American universities, and 99% were at universities in Western countries, including English-speaking countries (such as the UK, Canada, Australia, and New Zealand) and Europe. Only 1% of first authors were from Asia. Similarly, 68% of the samples were in the United States, 95% were in Western countries, and only 3% were in Asia. In other words, the other 95% of the world’s population is neglected. Henrich et al. (2010) further revealed that 96% of the subjects of psychological research published in the world’s top journals came from WEIRD societies, comprising only 12% of the world’s population. Raffaelli et al. (2013) contended that although a great majority of young people live in the “majority world” like developing countries, most contemporary theories and knowledge about adolescent development stemmed from research undertaken in the “minority world” such as WEIRD societies. Consequently, it is widely believed that psychological research depends heavily on WEIRD samples and among those from university students (Pollet and Saxton, 2019), which is incomplete and does not sufficiently represent the whole humanity.

In view of such phenomenon, three points are worth noting. First, although WEIRD samples included European participants, most researchers assumed that there should be many similarities between the United States and the rest of the West; consequently, few empirical studies sought to contrast Americans with European samples (Henrich et al., 2010). Second, even in the United States, research subjects are frequently biased toward middle-and upper-class children (Henrich et al., 2010), neglecting minorities and Asian Americans who might carry their CHC’s upbringing. Third, due to the rise of globalization, a lot of international students with Confucian heritage culture are enrolled at American universities; at the same time, more and more international students attend universities in CHC’s countries. However, research on the cross-population comparisons remains scant. Meadon and Spurrett (2010) contended that, the remedy for the existing bias is to foster research capacity in the non-Western world. Building research capacity should aim to generate studies led and initiated by non-Western researchers, who not only bring novel perspectives and ideas and are less affected by WEIRD bias, but also study non-WEIRD subjects, thus deepening the subject pool. This research provided a case in point, as it was initiated by non-WEIRD researchers with non-WEIRD samples.

Dweck et al.’s implicit theories (1988) and Rattan et al.’s (2012) findings originated from WEIRD nations may not fully apply to non-WEIRD societies such as CHCs. We should be extremely cautious to directly implant Western theories or research in non-Western cultural contexts. We argued that while some phenomena seem to be culture-free, others might be culture-bound. It is important for non-Western researchers to differentiate between the phenomena that are universal and culture-specific. The current findings showed that while ability-based paradigm might be culture-free, duty-based paradigm seems to be culture-bound. In both the West and CHCs, teachers who endorsed entity theory tended to give ability-comforting feedback. When students received such feedback, they tended to perceive their teacher as having entity belief and attributing failure to low ability as well as reported pursuing non-math related fields. As to the culture-bound paradigm, teachers who held culturally relevant obligation belief are more likely to offer duty-comforting and duty-advising feedback. Students responding to such feedback tended not only to perceive their teacher as having obligation belief and attributing failure to insufficient duty fulfillment, but also decided to stay on.

### Student perceptions of hidden messages in teacher feedback

Past research showed that teacher emotions like sympathy or pity (Graham, 1984; Butler, 1994; Georgiou et al., 2002; Taxer and Frenzel, 2020) and teacher behaviors like praise following success at easy tasks or the absence of blame at such task (Barker and Graham, 1987; Graham and Barker, 1990; Weiner, 2010) can indirectly and even unknowingly convey low-ability cues. Rattan et al.’s (2012) research further found that the well-intentioned ability-comforting teacher feedback not only communicated low ability cue and teacher’s entity belief, but also led struggling students to give up on math. Students’ perceptions of hidden messages in ability-comforting feedback were the attribution of their failure to low ability, which is fixed and uncontrollable. Since there is nothing they can do about it, they may feel hopeless and pessimistic about pursuing a career in math related areas thus change course.

Echoing Rattan et al.’s study, our research extended the finding that students perceived the messages underlying duty-comforting and duty-advising feedback conditions as their teacher’s obligation belief and cues of insufficient duty fulfillment. Since duty fulfillment is a controllable and obligatory factor, as long as they exert themselves to fulfill their obligations by working hard or working smart, their math ability can be developed and enhanced through continuous self-perfection. Instead of pursuing non-math related fields, they tended to stay on and try to overcome the difficulties and challenges during math learning. By doing so, they still have a ray of hope for success and cultivate their moral virtues.

### Trade-off between positive and negative dimensions of ability-comforting vs. duty-comforting feedback

Although both ability-comforting and duty-comforting feedback tend to reduce students’ pressure and discomfort, each type of comfort has its positive and negative sides. Ability-comforting feedback sends a message that while some are math persons, others aren’t, implying that math persons succeed without persevering through difficulties or challenges. Such feedback functions as low ability cues that failing students have no talent for math, which pushes them to switch to other non-math related fields, causing potential decrease in the talent pool of STEM students; however, these students might have greater opportunities to develop their full potentials and maintain their positive self-esteem in non-math related fields.

Duty-comforting feedback also has its bright and dark sides. Previous research revealed that, in CHCs such as Taiwan, compared with the arts, math was more likely to meet high parental expectation, students’ greater sense of obligation and stronger peer competition (Fwu et al., 2017). Furthermore, it is widely believed that math is a “critical filter” for admission to top universities and STEM degrees, which lead to higher-paying jobs. Duty-comforting feedback sends a message that as long as one has exerted oneself in the learning process, one does not owe anyone an apology. Such feedback encourages students to persist to overcome difficulties and maintain their engagement in math learning. This may be one of the reasons why East Asian students perform well on international assessments such as PISA and TIMSS (Schleicher, 2019; Mullis et al., 2020), and students in Taiwan tend to pursue “hot fields” such as engineering, science and medicine (Guo, 2013), providing a deep talent pool for tech giants like TSMC, which produces the majority share of the world’s semiconductor chips. However, the high math and science achievement of CHC’s students on PISA is accompanied by higher levels of anxiety and self-doubting (Wilkins, 2004; Lee, 2009). This phenomenon may be related to the pressure to continuously do well on examinations among CHC’s students (e.g., Tan and Yates, 2011). This concurred with previous findings that struggling students holding obligation-oriented belief about effort still tended to make effort after academic failure (Fwu et al., 2018). On top of this, duty-comforting feedback also conveyed a message that even though students are not good at math, they are still discouraged to change course. Under such circumstances, struggling students are under great pressure to stay on even when they face repeated math failures, which may be possible reasons to explain why students in CHCs suffered more mental health issues than their international counterparts (Lee, 2009; Morony et al., 2013; Stankov, 2013; Liu et al., 2017).

### Practical implications of conflicting feedback

Our research found that, when encountering students’ academic setbacks, a majority of teachers tended to provide ability-comforting, duty-comforting, and duty-advising feedback concurrently. What would be the reason for such mixed and contradictory feedback? It could be that teachers are influenced by both traditional obligation belief and recent Taiwanese education reforms, including 12-year basic education for all, and the new 12-year curriculum guidelines since the 2010s. Drawing inspiration mainly from WEIRD societies such as the United States, Finland, New Zealand, and the UK, the education reforms aimed to provide students with adaptive education to unleash their full potential, and broaden the scope of school success in both academic and non-academic domains in the process of student learning (Fwu et al., 2017; Coudenys et al., 2022). In addition to traditional duty-comforting and duty-advising feedback, we suggested that under the influence of recent education reforms, teachers were likely to provide ability-comforting feedback to encourage failing students to pursue the fields they believe they can develop their full potential.

The co-existence of conflicting feedback was likely to put students in a confusing and awkward situation that might be detrimental to their psychological well-being. The present research connects with other lines of research contending that CHC’s students were often torn between moving forward and pulling back in academic learning. For instance, prior research revealed that low-performing students are trapped in a dilemma between a distressing emotional state (“feeling bad”) for making too much effort in vain and a negative image (“being bad”) for not making enough effort to fulfill their duty (Fwu et al., 2017). Moreover, failing students experience both the de-motivating emotion of hopelessness, discouraging them from trying to do well, and the motivating emotion of indebtedness, triggering persistence. These conflicting emotions create a predicament for students—whether to work hard or not (Fwu et al., 2021). All these dilemmas students suffer may reinforce their psychological distress derived from academic stress. We suggest that it is not only failure but also the constant forces of pushing forward and pulling back after failure from various perspectives that contribute to poor psychological well-being of CHC’s students.

### Limitations and future research

This research has several limitations. First, our samples were limited to public “star” senior high schools and universities in the Taipei metropolitan area of northern Taiwan. Whether these results can extend to students in other schools and universities in Taiwan, as well as in other CHCs and Western societies would be an interesting question for future research. Second, the present research found that students exposed to different feedback conditions demonstrated differences in students’ perceptions about their teacher’s belief and attribution and behavioral responses. Future studies should investigate whether students’ own belief influences how they perceive teachers’ feedback and unveil the psychological mechanism in which students’ perceptions of teacher feedback relate to how they respond to feedback. Third, our research revealed that CHC’s teachers tend to provide failing students with different combinations of teacher feedback. Further research is needed to identify what factors, e.g., teacher belief or teacher attribution, and how these factors affect teacher feedback following students’ math failure.

## Conclusion

Current mainstream psychological theories and research findings derived from WEIRD societies have been so dominant in academic circles that non-WEIRD researchers unconsciously transplant them to their native societies without a doubt (Hwang, 2005, 2010). Findings stemming from such approach are mostly irrelevant to or inadequate for understanding the mentalities of local populations (Sinha, 1986). This research illustrated that it seems difficult to explain the dilemmas that CHC’s students encountered without considering cultural factors that may influence teacher feedback and student responses. Therefore, non-WEIRD scholars are advised not only to be skeptical about the theories and findings derived from WEIRD samples but also to further distinguish between culture-universal and culture-specific phenomena in advancing psychological knowledge.

## Data availability statement

The original contributions presented in the study are included in the article/Supplementary material, further inquiries can be directed to the corresponding author.

## Ethics statement

The studies involving human participants were reviewed and approved by Research Ethics Committee, National Taiwan University. Written informed consent for participation was not provided by the participants’ legal guardians/next of kin because: Our research has obtained IRB approval from National Taiwan University (NTU-REC: 201705HS032). In accordance with the protocol outlined by NTU-REC: 201705HS032, no parental consent was required.

## Author contributions

B-JF wrote the manuscript drafts, developed the study concept, and participated in designing the study and interpreting the findings. T-RY contributed to formulating the research questions, structuring the study design, organizing the analysis, and interpreting the results. Y-KC contributed to organizing and performing the statistical analysis, and participated in structuring the study design and interpreting the findings. RC revised the final manuscript. All authors read and approved the submitted version. All authors contributed to the article and approved the submitted version.

## Funding

This research is based upon work supported by a grant awarded to the first author, B-JF, under Award No. MOST 110-2410-H-002-080-SS3 from the National Science and Technology Council in Taiwan (https://www.nstc.gov.tw/). The funder had no role in study design, data collection and analysis, decision to publish, or preparation of the manuscript.

## Conflict of interest

The authors declare that the research was conducted in the absence of any commercial or financial relationships that could be construed as a potential conflict of interest.

## Publisher’s note

All claims expressed in this article are solely those of the authors and do not necessarily represent those of their affiliated organizations, or those of the publisher, the editors and the reviewers. Any product that may be evaluated in this article, or claim that may be made by its manufacturer, is not guaranteed or endorsed by the publisher.

## Supplementary material

The Supplementary material for this article can be found online at: https://www.frontiersin.org/articles/10.3389/fpsyg.2022.1046806/full#supplementary-material
