# Supplementary material for: The impact of teacher feedback on students’ decisions to stay on or change course after math failure in a Confucian cultural context
Source: Front Psychol. 2022 Dec 7;13:1046806. doi: 10.3389/fpsyg.2022.1046806 (PMC9768193; doi:10.3389/fpsyg.2022.1046806)
Supplement: Supplementary file 1 [file Data_Sheet_1.docx]

**Supplementary Material 1: Scale Items and Reliabilities**

**Study 1**

**Teacher Entity Belief (EB) Subscale** (α = .88, 4 items)

I think that

everyone is a certain kind of person, and there is not much that can be done to really change that (EB1).

people can do things differently, but the important parts of who they are can’t really be changed (EB2).

people can’t really change their deepest attributes (EB3).

the kind of person someone is, is something very basic about them and it can’t be changed very much (EB4).

**Teacher Obligation Belief (OB) Subscale** (α = .90, 5 items)

I think that

one should be ever-seeking to improve and further refine oneself (OB1).

it’s everyone’s responsibility to constantly perfect oneself to become a better self (OB2).

striving for further progress endlessly is a duty as a human being (OB3).

one is obligated to constantly improve oneself (OB4).

it’s one’s duty to make continual progress every day (OB5).

**Teacher Attribution to Lack of Ability (LA) Subscale** (α = .88, 3 items)

I think that

Minghua is not good at the subject I taught (LA1).

Minghua is not born with a talent for the subject I taught (LA2).

Minghua has no innate ability for the subject I taught (LA3).

**Teacher Attribution to Lack of Duty Fulfillment (LD) Subscale** (α = .90, 4 items)

I think that

Minghua did not exert his/her utmost effort to fulfill the role obligation as a student (LD1).

Minghua did not do whatever he/she can to fulfill the responsibility (LD2).

Minghua did not try as hard as he/she can to fulfill the duty (LD3).

Minghua did not do his/her very best as a student (LD4).

**Ability-comforting (AC) Feedback Subscale** (α = .82, 8 items)

It’s just not the case that everyone has a talent for this subject (AC_1).

Not everyone can be good at the subject (AC_2).

Not everyone needs to perform well in this subject (AC_3).

Not everyone is born with a talent for the subject (AC_4).

I will try not to call you to answer questions in class to ease your stress (AC_5).

I will give you fewer assignments so you can get more comfortable (AC_6).

I’m going to give you some easier tasks to work on to lessen your frustrations (AC_7).

I will try not to call you to answer questions on the blackboard in class to avoid putting you on spot (AC_8).

**Duty-comforting (DC) Feedback Subscale** (α = .82, 7 items)

Although you did not perform well, what matters is your attitudes in the learning process (DC_1).

Good attitude in the learning process is more important than end results (DC_2).

Making effort is the most important part of the learning process (DC_3).

Never giving up in the learning process is more important than the exam results (DC_4).

If you have difficulty understanding the learning materials, you can ask your classmates for help (DC_5).

You can learn study strategies from high-achieving peers (DC_6).

If you have problem understanding what was taught, you can ask me after class (DC_7).

**Duty-advising (DA) Feedback Subscale** (α = .89, 8 items)

You will always harvest what you plant. As long as you make effort, you will get positive outcomes (DA_1).

No pains, no gains. If you don’t work hard, there won’t be good results (DA_2).

There is no free lunch. Hard work will finally pay off (DA_3).

You should spend more time on study (DA_4).

You had better put more energy into this subject (DA_5).

You should review what was taught in class (DA_6).

You need to do more exercises (DA_7).

You had better start preparing for the exam early (DA_8).

**Study 2**

**Perceived Entity Belief (EBp) Subscale** (α = .90, 4 items)

After hearing teacher feedback, I felt that my teacher believed

everyone is a certain kind of person, and there is not much that can be done to really change that (EBp1).

people can do things differently, but the important parts of who they are can’t really be changed (EBp2).

people can’t really change their deepest attributes (EBp3).

the kind of person someone is, is something very basic about them and it can’t be changed very much (EBp4).

**Perceived Obligation Belief (OBp) Subscale** (α = .94, 4 items)

After hearing teacher feedback, I felt that my teacher believed

One should be ever-seeking to improve and further refine oneself (OBp1).

It’s everyone’s responsibility to constantly perfect oneself to become a better self (OBp2).

Striving for further progress endlessly is a duty as a human being (OBp3).

One is obligated to constantly improve oneself (OBp4).

**Perceived Attribution to Lack of Ability (LAp) Subscale** (α = .94, 3 items)

After hearing teacher feedback, I felt that my teacher thought

I’m not good at math (LAp1).

I am not born with a talent for math (LAp2).

I am not a math person (LAp3).”

**Perceived Attribution to Lack of Duty Fulfillment (LDp) Subscale** (α = .96, 4 items)

After hearing teacher feedback, I felt that my teacher thought

I did not exert my utmost effort to fulfill the role obligation as a student (LDp1).

I did not do whatever I can to fulfill the responsibility (LDp2).

I did not try as hard as I can to fulfill the duty (LDp3).

I did not do my very best as a student (LDp4).

**Staying-on Subscale** (α = .80, 3 items)

After I heard teacher feedback, I felt

I want to work hard to improve my math grades (SO1).

I am motivated to improve my math performance (SO2).

I don’t give up easily in the face of difficulty on math tests (SO3)*.*

**Path-changing Subscale** (α = .91, 4 items)

After I heard teacher feedback, I thought

I will switch to a field that I am good at (PC1).

I will put effort into other goals that I am interested in (PC2).

I will shift to a direction that I can develop my full potentials (PC3).

I will change to the areas I have talents for (PC4).

**Supplementary Material 2: Three Conditions of Teacher Feedback in Study 2**

**Ability-comforting (AC) Feedback**

“I know you are a talented student in general. It’s just not the case that everyone is a ‘math person.’ I want you to remember how great you do in other subjects. I want you to know that I’m not going to call on you as much in math class because I don’t want you to have pressure and I’m going to give you some easier math tasks to work on so you can feel more comfortable. I want to assure you that I really care, so let’s stay in contact about how you’re doing in the class.”

**Duty-comforting (DC) Feedback**

“I know you are a dedicated student in general. Although you didn’t get good grades, don’t feel upset. The only thing that matters is the learning attitude that you have done your best. If you have trouble understanding math, we can discuss why you didn’t do well on the exam after class or you can find a classmate who can help you with math. I want to assure you that I really care, so let’s stay in contact about how you’re doing in the class.”

**Duty-advising (DA) Feedback**

“I know you are a dedicated student in general. No pains, no gains. If you did not try your best to study math, you would certainly not get good grades. I hope you study harder and spend more time on math. I suggest you start preparing for the exam earlier and do more math exercises. I want to assure you that I really care, so let’s stay in contact about how you’re doing in the class.”

| **Supplementary Material 3**  *Descriptive Statistics for All Items (N = 270)* | | | | | |
| --- | --- | --- | --- | --- | --- |
| Name | Items | Mean | *SD* | Skewness | Kurtosis |
| EBp | EBp1 | 4.27 | 1.06 | -0.28 | -0.43 |
|  | EBp2 | 4.22 | 1.03 | -0.24 | -0.43 |
|  | EBp3 | 4.25 | 1.01 | -0.41 | -0.01 |
|  | EBp4 | 4.25 | 1.06 | -0.43 | -0.17 |
| OBp | OBp1 | 3.21 | 1.39 | 1.85 | 11.53 |
|  | OBp2 | 3.41 | 1.15 | 0.17 | -0.37 |
|  | OBp3 | 3.29 | 1.23 | 0.16 | -0.70 |
|  | OBp4 | 3.03 | 1.14 | 0.27 | -0.42 |
| LAp | LAp1 | 3.54 | 1.57 | 0.02 | -1.14 |
|  | LAp2 | 3.17 | 1.50 | 0.34 | -0.95 |
|  | LAp3 | 3.27 | 1.58 | 0.24 | -1.06 |
| LDp | LDp1 | 3.44 | 1.61 | 0.11 | -1.27 |
|  | LDp2 | 3.61 | 1.55 | -0.06 | -1.18 |
|  | LDp3 | 3.65 | 1.43 | -0.11 | -0.91 |
|  | LDp4 | 3.61 | 1.54 | -0.05 | -1.06 |
| Staying-on | SO1 | 4.13 | 1.72 | 4.50 | 46.45 |
|  | SO2 | 4.64 | 1.05 | -1.15 | 2.07 |
|  | SO3 | 4.14 | 1.18 | -0.54 | 0.38 |
| Path-changing | PC1 | 4.44 | 0.99 | -0.70 | 1.31 |
|  | PC2 | 3.28 | 1.29 | 0.09 | -0.36 |
|  | PC3 | 3.35 | 1.17 | 0.13 | -0.06 |
|  | PC4 | 3.51 | 1.20 | -0.16 | -0.16 |
| *Note*. EBp = perceived teacher’s entity belief; OBp = perceived teacher’s obligation belief; LAp = perceived teacher’s attribution to lack of ability; LDp = perceived teacher’s attribution to lack of duty fulfillment; SO = Staying-on; PC = Path-changing. | | | | | |

**Supplementary Material 4**

To conduct LCA, we randomly divided all items of AC, DC and DA feedback subscales in Study 1 into two parts that were labelled as AC1 and AC2, DC1 and DC2, as well as DA1 and DA2. We then transformed the 6-point scales into dichotomous variables using a cut-off score of 3.5.

AC1: mean of AC_1, AC_2, AC_5, and AC_6.

AC2: mean of AC_3, AC_4, AC_7, and AC_8.

DC1: mean of DC_1, DC_2, DC_5, and DC_6.

DC2: mean of DC_3, DC_4, and DC_7.

DA1: mean of DA_1, DA_4, DA_5, and DA_6.

DA2: mean of DA_2, DA_3, DA_7, and DA_8.
